# Polyethylene glycol-based isolation of urinary extracellular vesicles, an easily adoptable protocol

**DOI:** 10.1016/j.mex.2023.102310

**Published:** 2023-08-06

**Authors:** Anula Divyash Singh, Sreekanth Patnam, Anisha Manocha, Leena Bashyam, Aravind Kumar Rengan, Manda Venkata Sasidhar

**Affiliations:** a Apollo Hospitals Educational and Research Foundation (AHERF), Hyderabad, India; b Department of Biomedical Engineering, Indian Institute of Technology Hyderabad (IITH), Kandi, Hyderabad, India; c Department of Histopathology, Apollo Hospitals, Hyderabad, India; d Genomics Facility, School of Life Sciences, University of Hyderabad, Hyderabad, India; e Urvogelbio Private Ltd, Hyderabad, India

**Keywords:** Isolation of urinary extracellular vesicles, Liquid biopsy, Non-invasive diagnostics, Polyethylene glycol, Renal pathologies, Urinary extracellular vesicles

## Abstract

Urine is a highly advantageous biological specimen for biomarker research and is a non-invasive source. Most of the urinary biomarkers are non-specific, volatile and need extensive validation before clinical adoption. Extracellular vesicles are secreted by almost all cells and are involved in homoeostasis, intercellular communication, and cellular processes in healthy and pathophysiological states. Urinary extracellular vesicles (UEVs) are released from the urogenital system and mirror the molecular processes of physiological and pathological states of their source cells. Therefore, UEVs serve as a valuable source of biomarkers for the non-invasive diagnosis of various pathologies. They hold a promising source of multiplex biomarkers suitable for prognosis, diagnosis, and therapy monitoring. UEVs are easily accessible, non-invasive, and suited for longitudinal sampling. Although various techniques are available for isolating UEVs, there is yet to be a consensus on a standard and ideal protocol. We have optimized an efficient, reliable, and easily adoptable polyethylene glycol (PEG) based UEV isolation technique following MISEV guidelines. The method is suitable for various downstream applications of UEVs. This could be a cost-effective, consistent, and accessible procedure for many clinical labs and is most suited for longitudinal analysis. Adopting the protocol will pave the way for establishing UEVs as the ideal biomarker source.

• Urine can be collected non-invasively and repeatedly, hence a very useful specimen for biomarker discovery. Urinary EVs (UEVs), derived from urine, offer a stable diagnostic tool, but standardised isolation and analysis approaches are warranted.
• To have enough UEVs for any study, large volumes of urine sample are necessary, which limits different isolation methods by cost, yield, and time.
• The protocol developed could help researchers by offering a cost-effective and dependable UEV isolation method and may lay the foundation for UEVs adoption in clinical space.

## Graphical abstract

**Figure:**
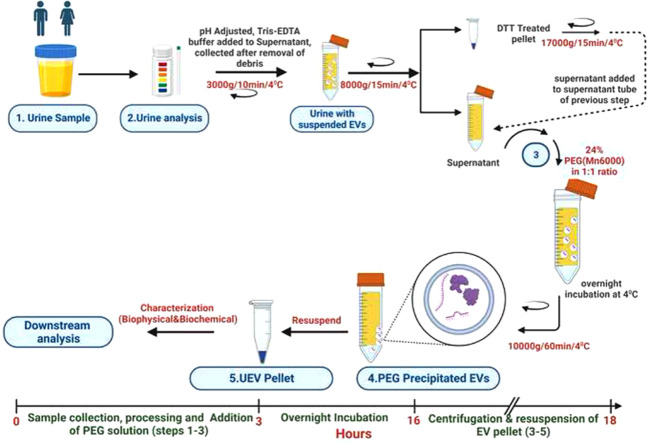
PEG-based isolation of urinary extracellular vesicles

Graphical abstract: This figure was created by using BioRender.com.

Specifications table

**Table:** 

Subject area	Biochemistry, Genetics and Molecular Biology
More specific subject area	*translational research and extracellular vesicles,*
Name of your method	*Isolation of urinary extracellular vesicles*
Name and reference of original method	*NA*
Resource availability	*NA*

## Method details

### Background

Extracellular vesicles (EVs) are constitutively secreted vesicles from all cells. EVs are bound by lipid bilayers and possess unique biosignatures of the source cells. EVs facilitate cell-to-cell communication by transporting various cargo, including proteins, lipids, DNA, and RNA, ranging from 30–1000 nm in size. EVs have been stratified into exosome-like vesicles (20–50 nm), exosomes (30–150 nm), membrane particles (50–80 nm), apoptotic bodies (50–500 nm) and microvesicles (100–1000 nm) [1]. EV subgroups vary enormously in size and biogenesis, however there is still considerable scepticism regarding their classification. As a result, the International Society of Extracellular Vesicles (ISEV) recommends referring to vesicles discharged from cells generically as "EVs." [2], [3], [4], [5]. Several messenger RNA (mRNA), micro-RNA (miRNA), and circulating miRNA are encased in urinary extracellular vesicles (UEVs), protecting them from degradation by other RNAases [6], [7], [8], [9]. There is growing evidence that urinary extracellular vesicles can be used as a source for biomarker discovery, diagnostics, prognostics, and therapeutic applications in a wide range of diseases, including renal disorders, kidney transplant and subsequent complications, diabetic kidney disease, renal cell carcinoma, and prostate cancer [3,[10], [11], [12], [13], [14], [15]]. The urine task force for extracellular vesicles has recently formulated recommendations for disclosing information regarding UEVs, including urine collection, storage, processing and UEV characterisation [16]. These recommendations aim to assist the rapidly expanding UEV-based research community. There is currently no consensus on the method for isolating urinary extracellular vesicles or the techniques for conducting analytical research on them. amongst the available methods for isolating UEVs are differential centrifugation, ultracentrifugation, hydrostatic filtration dialysis, commercial kit-based isolation methods, magnetic-beads isolation method, and nanomaterial-based isolation techniques [3,12,[17], [18], [19], [20], [21], [22]]. However, the above isolation methods are limited by their yield, purity, repeatability, cost, time consumption, and the need for larger urine volumes. Additionally, the downstream application of UEVs, such as transcriptomics, proteomics, or extracellular vesicle engineering, influences the selection of the isolation methodology. A highly scalable and cost-effective method would be beneficial for the scientific community. Scalability implies the capacity to accommodate larger urine volumes and increased sample sizes. Cost-effectiveness refers to the method's low operating expenses [22,23]. Summing up all the above aspects, we have optimized the UEVs separation process based on the PEG precipitation [19,24]. This method may facilitate the management of large volumes of urine and be used for longitudinal analysis of UEVs. This straightforward laboratory protocol employs the same water-excluding polymer principle as commercial kits [25]. It was created to provide a cost-effective method to extract urinary extracellular vesicles.

## Methodology

The Apollo Hospitals Internal Ethics Committee (IEC) approved the study with protocol number CMBRC/2014/005 and IEC application number 114.

### Urine collection and processing

1. After obtaining informed consent, urine samples from healthy individuals were collected in a sterile container (Abdos Lifescience, WB, India). The demographic characteristics are shown in Table 1.

**Table 1:** Demographics of Healthy Individuals

Parameter	value
Sample Size (N)	40
Age	47.2 ± 13.4
Gender	
Male (N)	35
Female (N)	5

Table 1 Age is represented as Mean ± SD.
2. Before sampling, 4.2 ml of protease inhibitor cocktail (1 mg/mL Leupeptin; G Biosciences, MO, USA), 10 mM sodium azide, and 50 mM PMSF (both from Sigma, MO, USA) were added to the collection container.
3. First-morning urine was collected and stored at 4 °C until processing.
4. Urine samples were brought to our laboratory and immediately processed for extracellular vesicle isolation using PEG-based precipitation. The collection and submission process for EV isolation takes around 15–60 min.
5. Complete urine examination (CUE) was performed at Apollo Diagnostics Lab, using urinalysis strips (Siemens Multistix 10 SG, Siemens, Munich, Germany) in the Clinitex Advantus instrument (Siemens) for an initial quality check of collected samples and to ensure urinary parameters were within normal range, as shown in Table 2.

**Table 2:** Summary of urine analysis of healthy individuals

Samples (40)	Leucocyte	Nitrite	Urobilinogen (mg/dl)	Protein (mg/dl)	pH	Blood	Specific Gravity	Ketone (mg/dl)	Bilirubin	Glucose (mg/dl)	Colour	Clarity
Observed	Negative	Negative	0.25	Negative	5.5–7.5	Negative	1–1.025	Negative	Negative	Negative	Pale Yellow	Clear
Reference range	Negative	Negative	0.2–3.2	Negative	5–8.5	Negative	1–1.03	Negative	Negative	Negative	Pale yellow	Clear

### Isolation of urinary extracellular vesicles

1. 20 mL of collected urine sample was centrifuged at 3000*g for 10 min at 4 °C to remove cell and cellular debris.
2. The supernatant was carefully transferred to a fresh tube; pH was adjusted to 4 using 0.2 N HCl, and 5 mL of Tris-EDTA (Sigma, MO, USA) buffer (20 mM pH 6.8) was added [26], [27], [28]. Appendix-2 represents overall pH values at each step of isolation process (Fig. 7).

**Fig. 7:**
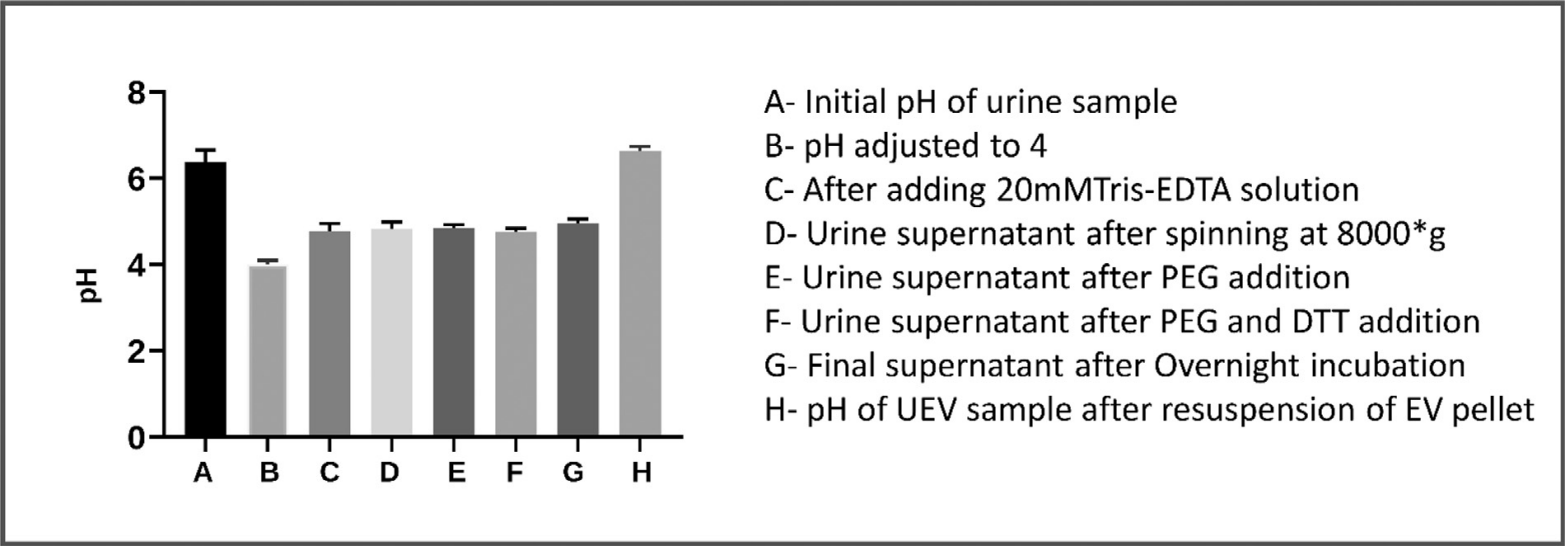
Sample pH at different stages of the isolation process. All data are represented as mean ± SD *n* = 3. The average pH range of the final resuspended UEV pellet is 6.62 ± 0.107.
3. The resultant supernatant mixture was vortexed for 90 s and centrifuged at 8000*g for 15 min at 4 °C. After the centrifugation step, the supernatant was collected in a fresh tube and thoroughly mixed with UEV precipitation solution [24% (w/v) PEG Mn6000 prepared in 1 M NaCl (Sisco Research Laboratories (SRL), Mumbai, India)] in 1:1 ratio with the processed urine supernatant, so the final working concentration of PEG is 12% w/v [28];
4. The pellet obtained in the above step was treated with 500 μL of 100 mM DTT (Sigma, MO, USA) and incubated for 10 min at 37 °C for Tamm-Horsfall Glycoprotein (THP) removal [29].
5. Subsequently, the DTT-treated UEV pellet was centrifuged at 17,000*g for 15 min at 4 °C with no brake. The supernatant obtained here was added to the previously obtained PEG- supernatant mixture from step 3 and incubated overnight at 4 °C [19].
6. The PEG-supernatant mixture was centrifuged at 10,000*g for 60 min at 4 °C the following day. Finally, the pellet containing UEVs was resuspended in 300 μL PBS and stored at −80 °C for future use.
7. The centrifuge used for all centrifugation steps was from Eppendorf model no.5810R; Rotor no. F-34–6–38 (Eppendorf, Hamburg, Germany).
8. The UEV isolation procedure is depicted as a flowchart in Fig. 1. The total time required for sample processing and UEV isolation is represented in Appendix-1 Fig. 6.

**Fig. 1:**
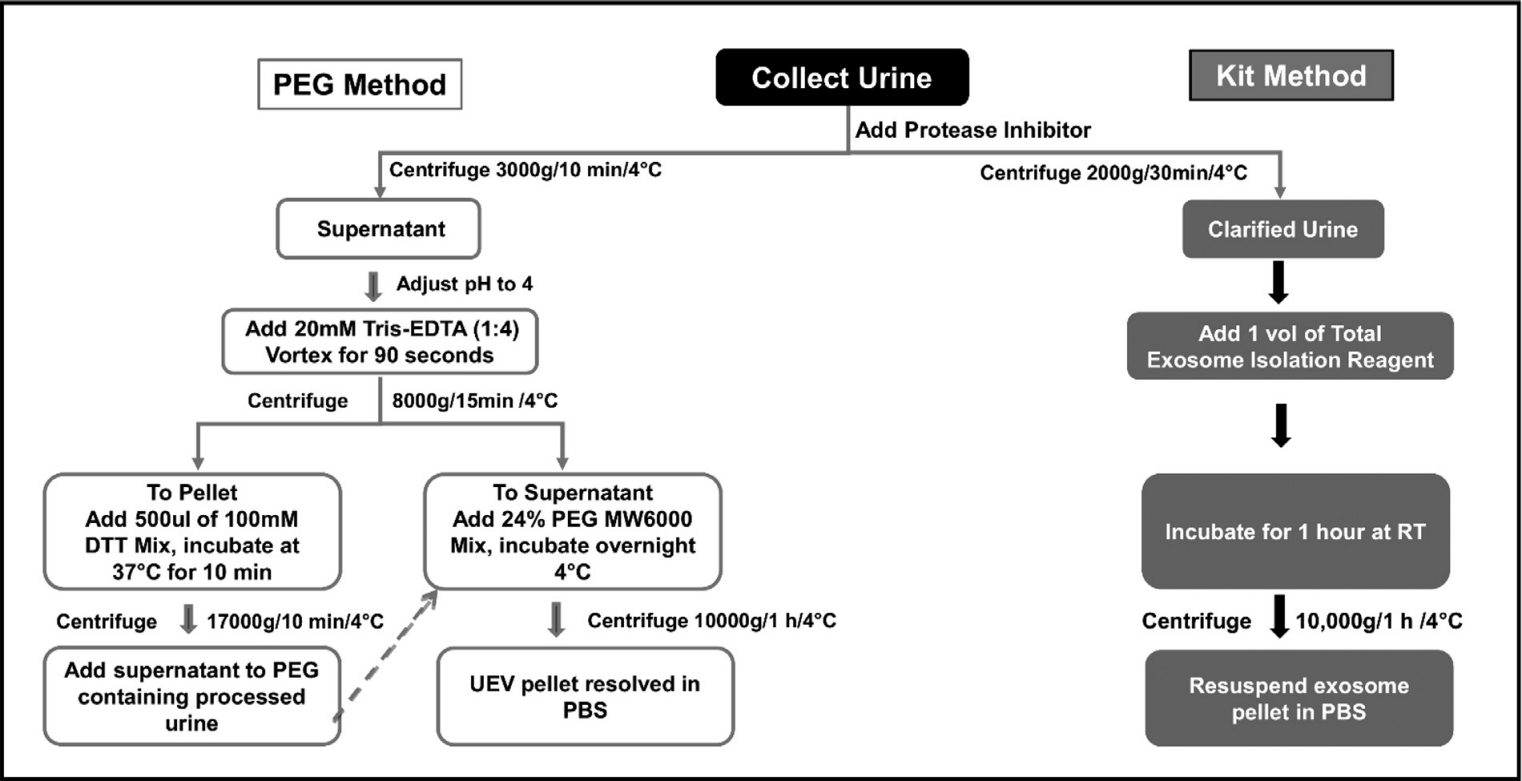
Schematic representation of the Urinary Exosome Isolation Procedure using PEG and commercially available kit method.

**Fig. 6:**
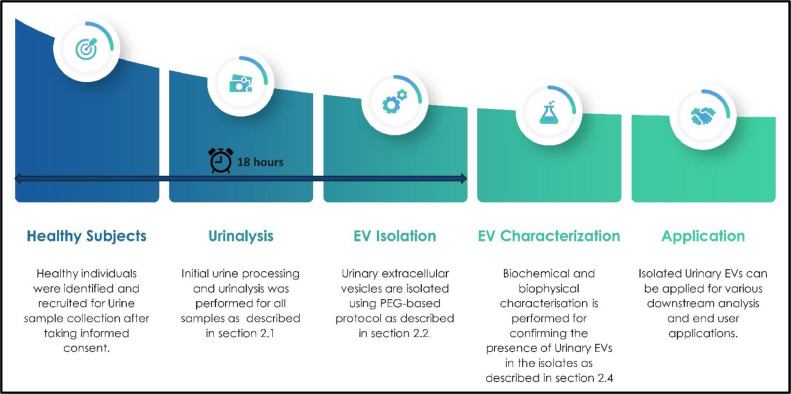
Representation of overall procedure with the time of processing of UEV isolation.

### Optimisation of extracellular vesicles isolation procedure

1. We optimised the isolation procedure by varying the PEG Mn6000 concentrations (6%, 12%, and 24% (w/v)). 12% (w/v) PEG (final concentration) yielded the maximum total protein content and extracellular vesicle yield (Fig. 2a–e).

**Fig. 2:**
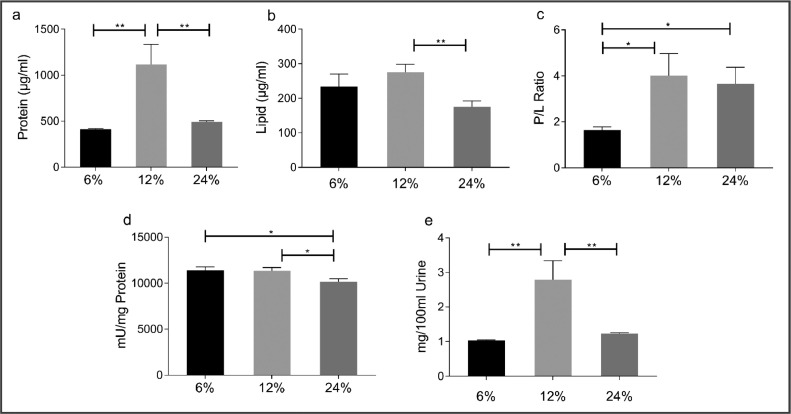
Biochemical properties with varying PEG Mn6000 concentrations. (A) One-way ANOVA comparison of the total protein content of UEVs extracted using various concentrations of PEG MW6000 (*n* = 3), *p* = 0.0009. (B) Lipid content of the isolated UEVs using Phospho Vanillin Assay, *p* = 0.0105 (C) Protein to Lipid ratio of the UEV, *p* = 0.0120 (D) Acetylcholinesterase assay of UEV confirms the presence of the exosome in UEV fraction, *p* = 0.086. (E) Total EV yield per 100 ml urine sample, *p* = 0.0009. All data are represented as Mean ± SEM, *n* = 3. Significance *P* ≤ 0.05 is represented by * and *P* ≤ 0.01 as **.
2. We also isolated urinary extracellular vesicles using a commercially available standard kit as a control and compared the biochemical properties of UEV fractions from both methods.
3. Quantitative analysis of the total protein, lipid content, protein-to-lipid ratio, and total yield using both methods revealed moderate differences, which are insignificant (Fig. 3a–d) [30].

**Fig. 3:**
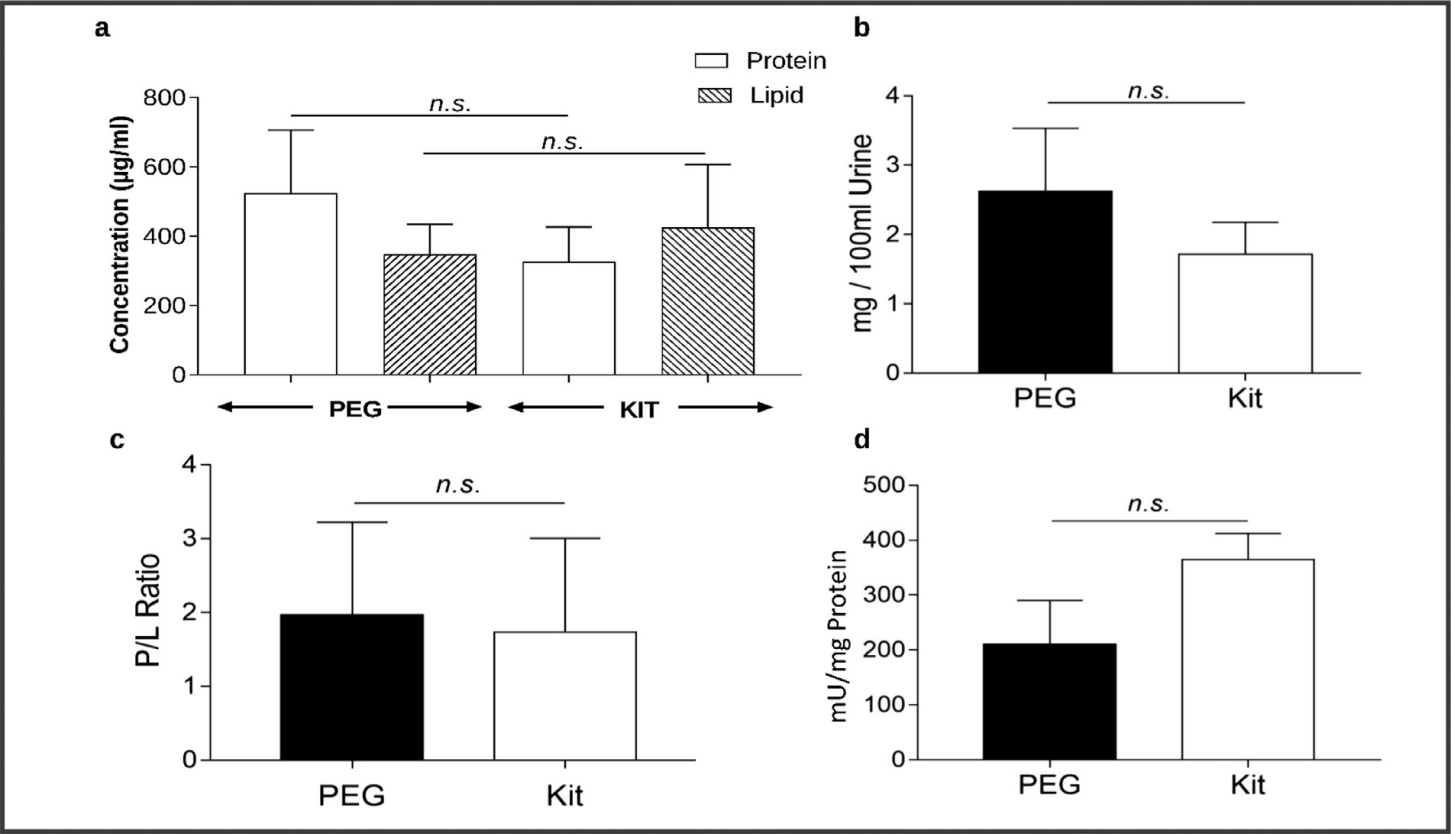
Biochemical properties and Comparison of UEVs isolated by PEG and Kit method. (A) Total protein and lipid content of the UEVs isolated. (B) Comparison of UEV yield per 100 ml of urine. (C) The ratio of Protein to Lipid contents is shown. (D) Acetylcholinesterase activity of the isolated UEV using both methods. The biochemical properties of the UEVs derived using both methods were comparable. All data are represented as mean ± SEM, *n* = 3.

### Urine extracellular vesicles characterisation

*Biophysical and biochemical characterisations were performed on the isolated UEVs to ensure the purity of the isolated EVs.*

#### Biochemical characterisation

**Fig. 4:**
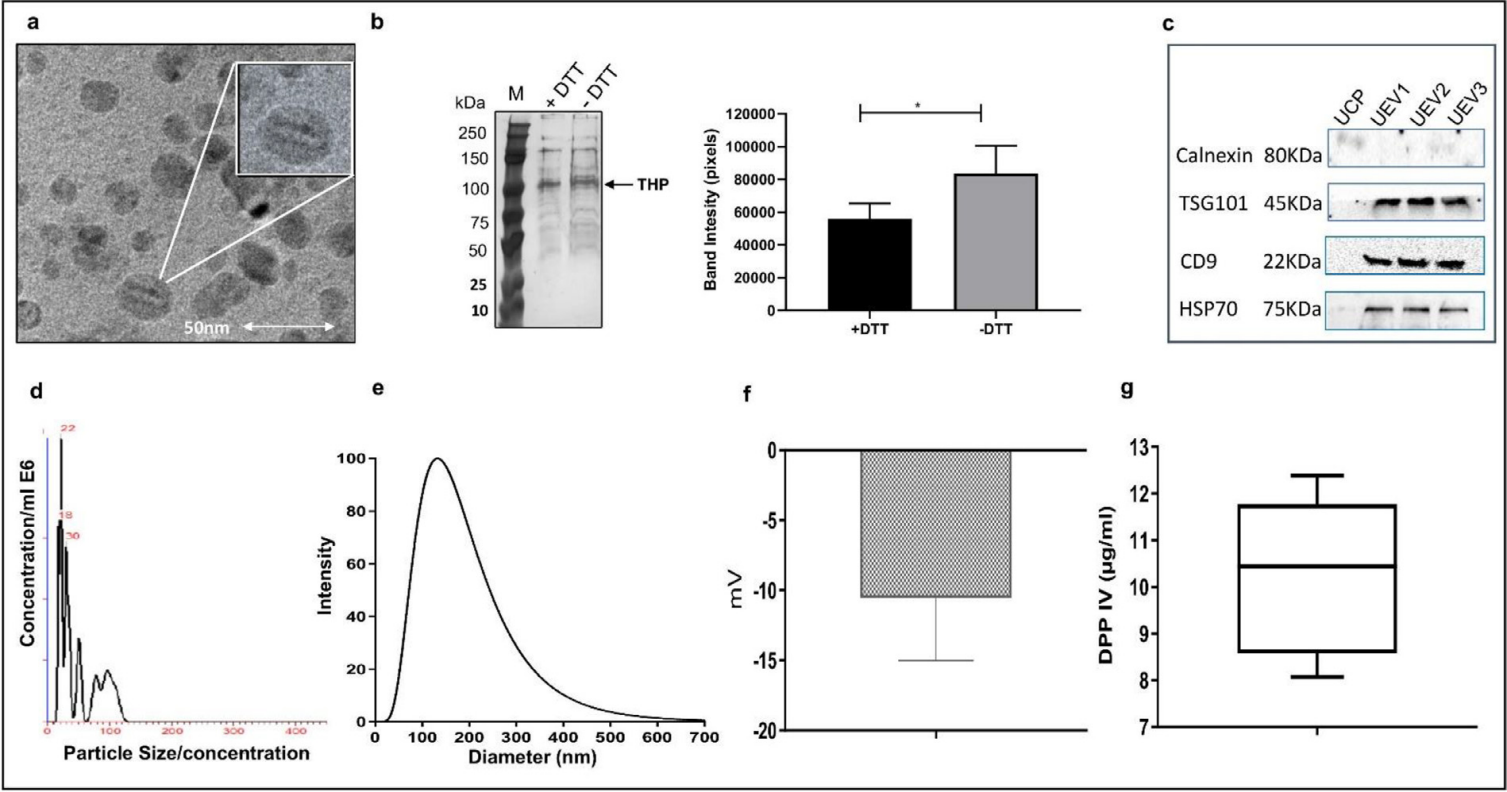
UEV characterisation, morphology, and size distribution. (A) Representative TEM images of the isolated UEVs are shown. The scale bar represents 50 nm (B) SDS-PAGE analysis confirmed the reduction of THP protein in the DTT-treated fraction compared to the untreated fraction. (C) Western blotting analysis confirmed the presence of EV-specific protein markers. UEV 1, 2 and 3 represent three independent urinary EVs preparation, and UCP is urinary cell pellet. (D) NTA analysis revealed the size of UEVs ranging between 30 and 130 nm. The average size from *n* = 3 acquisition was 57.3 ± 8.38 nm, and the concentration was 3.85 ± 1.3 E8 particles/mL. (E) Size distribution of UEV isolates measured by DLS is shown as an intensity distribution curve with a mean particle diameter of 160.5 ± 18.79 nm. (F) Vesicle membrane potential was determined by zeta potential, which was −10.5 ± 4.48 mV. (G) Dipeptidyl peptidase IV activity confirms the UEV isolates as the microvesicular component of urine. Data illustrated in d-G are represented as mean ± SD, *n* = 3.

*Protein estimation and SDS-PAGE analysis:* The total protein content in the UEV isolate was quantified using bicinchoninic acid (BCA) protein assay kit (G Biosciences, MO, USA) following the manufacturer's instructions (Fig. 3a).  For SDS-PAGE, 50 μg of UEV protein were lysed using RIPA lysis and extraction buffer (G Biosciences, MO, USA), including protease inhibitor cocktail (Roche, Basel, Switzerland), followed by incubation at 4 °C for 30 min. The lysed protein samples were mixed with reducing Laemmli sample buffer and denatured for 10 min at 70 °C. 50 μg of UEV protein was resolved on 10% SDS PAGE for 1.5 h at 120 V, and the gels were stained using 0.2% silver nitrate (Fig. 4b) [31].

*Lipid estimation:* The total lipid content of the UEV isolates was quantified using the phosphovanillin assay, as previously reported [32]. Briefly, 200 µl of 96% H_2_SO_4_ was added to 40µL of the lipid standard, DOPC (1,2-Dioleoyl-sn‑glycero-3-phosphocholine, Avanti Polar lipids Inc., AL, USA), or the UEV samples and evaporated at 90 °C on a dry bath for 20 min. After the tubes were cooled to RT, 120 µL of phosphovanillin reagent (50 mg of vanillin dissolved in 50 mL of 17% H_3_PO_4_; Sigma, MO, USA) was added. 180 µL of the resultant reaction mixture of standards and samples was transferred to respective wells in a 96-well plate and incubated for 1 h at 37 °C. The absorbence was recorded at 540 nm, and the total lipid was determined from the standard curve (Fig. 3a).

*Acetylcholinesterase (AChE) activity:* The presence of acetylcholinesterase is regarded as a marker enzyme for extracellular vesicles, was determined using a colorimetric enzyme assay [33]. 20 μL UEV fraction was added to a 96-well flat-bottomed microplate. 1.25 mM of Acetylthiocholine Iodide and 0.1 mM of 5′, 5′-dithio-bis (2-nitrobenzoic acid) (both from Sigma, MO, USA) were added to each well to a final volume of 300 μL. The absorbence was recorded at 412 nm every 5 min for 30 min. The AChE activity in the UEV fraction was determined from the AChE enzyme standard curve (Fig. 3d).

*Dipeptidyl peptidase-IV Activity:* The membrane-associated dipeptidyl peptidase IV (DPPIV), secreted by the kidney's tubular epithelial cells, is a component of urinary microvesicles associated with renal complications. As an additional criterion for assessing the UEVs, we used a colorimetric assay previously used to determine DPPIV activity in the serum [34]. Briefly, 50 μL of 71 mmol/L glycine/NaOH (pH 8.3) buffer and 10 μL of UEV sample were mixed in a 96-well plate. All wells, including the blank wells, received 50 μL of the 0.5 mg/mL substrate Gly-Pro-p-nitroanilide (Sigma, MO, USA), which was then incubated for 60 min at 37 °C. DPPIV in the sample disintegrates down the substrate, releasing free 4-nitroaniline, a chromogenic substance whose absorbence is measured at 405 nm in a plate reader. The DPPIV activity in the UEV sample was calculated against the standard plot of the p-nitroaniline standard. (Sigma, MO, USA) (Fig. 4g).

#### Morphological characterisation

*Transmission electron microscopic analysis (TEM):* TEM was performed to confirm the size and morphology of UEVs in the isolated fraction. EVs are spherical and cup-shaped vesicles [35]. Briefly, 10 μL of UEV isolates were fixed for 5 min with 1% glutaraldehyde on 400-mesh copper grids (FCF400-Cu, Electron Microscopy Sciences, Hatfield, PA). The grid was washed twice with water, stained with 2% uranyl acetate, and dried at room temperature using natural airflow. Transmission electron microscope (JEM-2100, JEOL Ltd., Tokyo, Japan) was used to capture images (Fig. 4a).

*Nanoparticle tracking analysis (NTA):* NTA involves tracking the Brownian motion of individual particles [36]. The size distribution within the UEV isolate was calculated using a nanoparticle tracking analyser (NanoSight, Malvern, Ltd., Malvern, UK). The measurement parameters were pre-set according to NTA instrument instructions. The measuring time was 30 s. 10 μL of the UEV fraction was diluted with PBS in the ratio of 1:1000 into 1 ml. The particle size and concentration of UEVs in the sample were detected and analysed by NTA software (version 2.3 Build 0025) (Fig. 4d).

*Dynamic Light Scattering (DLS):* DLS with zetasizer analysis was used to confirm the particle size and membrane potential of UEVs. The Brownian motion of the dispersed UEVs is used to measure their size in the dynamic light scattering analysis [37]. About 10 μL of UEV fractions were diluted in PBS (1:100) and made up to 3 ml to form a homogenous suspension. This was then dispensed into the cuvette, and the hydrodynamic diameter of UEV isolates was measured with the DLS instrument (Nicomp Nano Z3000, Entegris, MA, USA). Intensity-weighted distribution curve is used to evaluate the size of vesicles. At least three independent aliquots were measured (Fig. 4e–f).

#### Molecular analysis

For molecular validation, UEV-specific protein markers were assessed by western blotting, and UEV-RNA transcript was analysed by RT-qPCR.

*EV Protein Marker Analysis:* Following MISEV recommendations, EV-specific marker proteins were identified by western blotting analysis of the UEV isolate [2]. To obtain the total protein, isolated UEVs were harvested in RIPA lysis buffer containing protease- and phosphatase-inhibitors (G Biosciences, MO, USA) and quantified by BCA assay. 50 μg UEV protein was loaded in 10% SDS-PAGE. The protein bands were then transferred onto a nitrocellulose membrane using a wet transfer unit at 70 Vs for 2 h at 4 °C. The membrane blots were saturated for 2 h at room temperature with 5% non-fat dry milk in TBST. Following blocking, the blots were incubated overnight at 4 °C with primary antibodies (Abcam, Cambridge, UK), at a ratio of 1:2000, followed by 2 h of incubation at room temperature with secondary antibodies (Abcam, Cambridge, UK), at a ratio of 1:6000. The blot was developed. Images were captured with the iBright7500 instrument (Thermo Fisher Scientific, California, USA) (Fig. 4c).

**Fig. 5:**
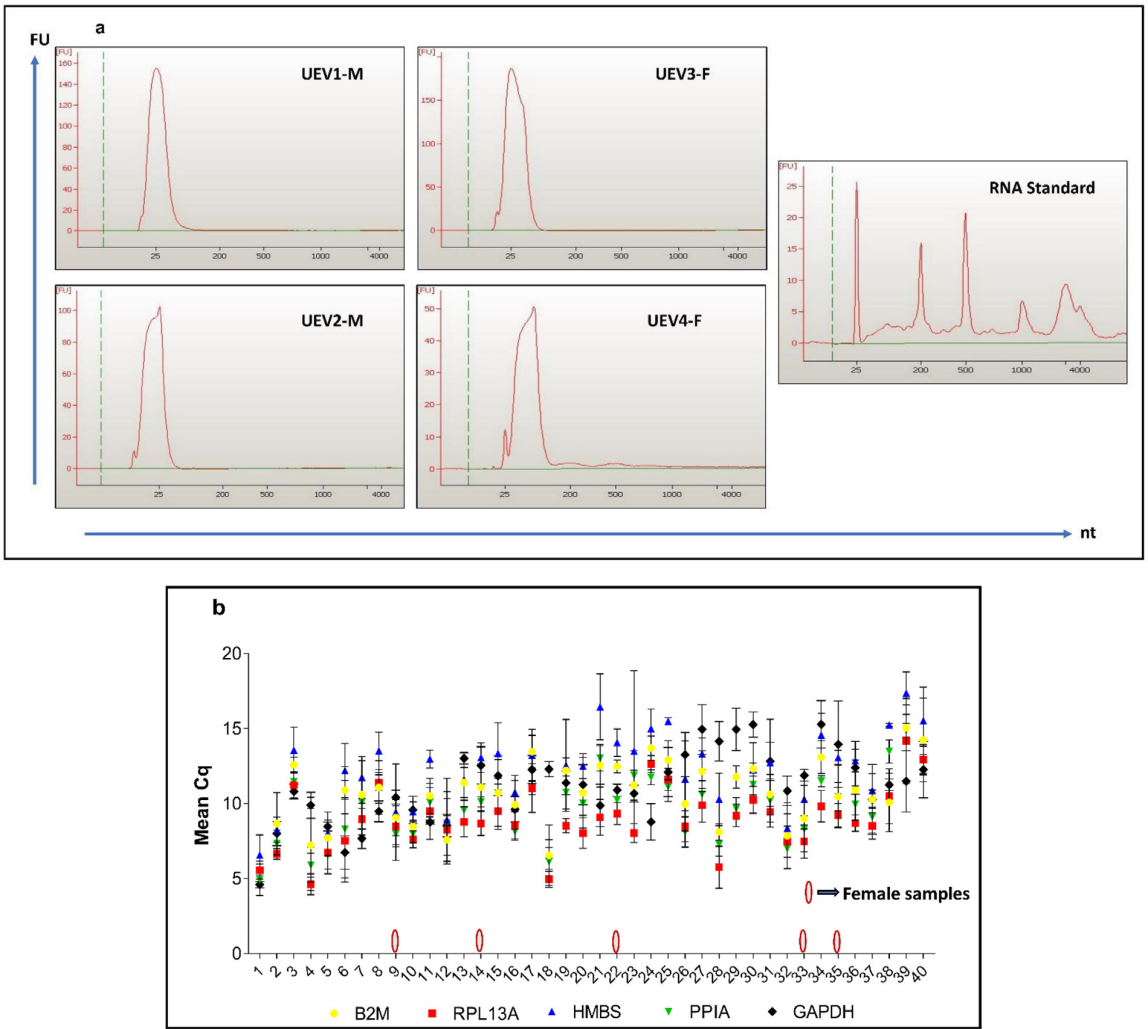
RNA analysis and RT-qPCR. (A) The RNA quality from UEVs was assessed using Agilent Bioanalyzer. Representative UEV RNA samples 2 from male (UEV1-M, UEV2-M) and 2 from female (UEV3-F, UEV4-F) individuals are shown with RNA standard profile. UEV-RNA can be observed as a sharp 5S RNA peak between 25 and 200 nt, while no detectable 18S and 28SRNA peaks were observed. The X-axis on the scale represents fluorescence units (FU), and the y-axis represents Nucleotide (nt). (B) Gene expression profiles of five housekeeping genes in UEVs are shown as quantification cycle (Cq) values. Female samples are marked in red. Datas are shown as SEM, *n* = 40.

*UEV RNA Extraction:* Total RNA from UEV isolates was extracted using TRIzol™ LS Reagent (Invitrogen, CA, USA) following the manufacturer's instructions. Summarising, the extracellular vesicle sample and Trizol reagent (1:3) were combined and incubated for 5 min at room temperature. The mixture received a 1:5 addition of chloroform, which was then centrifuged at 12,000*g for 15 min at 4 °C. The upper aqueous phase was carefully transferred to a fresh tube. An equal volume of isopropanol and 1 μL of 500 μg/mL Glycogen (GlycoBlue™ co-precipitant Invitrogen, CA, USA) were added to each tube and incubated at −20 °C overnight. The next day, the samples were centrifuged at 12,000*g for 15 min at 4 °C, and the pellet was washed with ethanol. The RNA pellet was air-dried for a few minutes, dissolved in nuclease-free water, and stored at −80 °C for further analysis. Qubit™ RNA HS Assay Kit was used to quantify the amount of RNA (Invitrogen, CA, USA). The UEV RNA was also analysed in the Agilent Bioanalyzer 2.1 instrument using an RNA Pico kit (both from Agilent Technologies, CA, USA) (Fig. 5a). The total RNA yield was 264 ± 83.8 pg/ml of urine. [38]

*Endogenous gene expression analysis:* RT-qPCR was used to confirm the expression of housekeeping genes in the UEV-RNA pool [39]. 1 μg of UEV-RNA obtained from the preceding step was reverse transcribed using a High-capacity cDNA Reverse Transcription kit (Invitrogen, CA, USA), following the manufacturer's instructions. Pre-amplified cDNA was diluted 1:5 with nuclease-free water, and 1 μL was used as a template for RT-qPCR. A 10 μL reaction was performed in the Applied Biosystems 7500 Real-Time System using 5 μL of TB Green Premix Ex Taq (Takara Bio Inc. Shiga Prefecture, Japan), 0.1 μL of a 10 μM primer pair, 1 μL of template cDNA, 3.8 μL of nuclease-free water, and a no template control. Real-time PCR was conducted for the 30 s at 95°C, followed by 35 cycles of 5 s at 95°C and the 30 s at 60°C. The cycle threshold (C_T_) values obtained for each housekeeping gene and the data are represented in Fig. 5b.

## Summary and conclusion

We demonstrated that the PEG-based precipitation method could efficiently isolate UEV at a lab scale. We confirm that the isolated EV fractions from the urine are majorly in the size range of exosomes. The recommended methodology produced comparable biochemical properties of UEVs with a commercial kit. A cost-to-benefit ratio with both methods would favour the lab-based protocol. Vesicle integrity was verified by their biochemical and biophysical characteristics. Protein markers unique to EVs were present to confirm their authenticity as per MISEV guidelines. The presence of housekeeping genes in mRNA transcripts of isolated UEVs was confirmed by gene expression analysis.

The proposed isolation method is highly cost-effective and feasible when dealing with larger sample sizes and higher volumes during longitudinal analysis. Extracting urinary extracellular vesicles (UEVs) with this method requires a basic bench-top centrifuge and proper storage conditions. Still, these requirements remain less demanding than methods like filtration, size exclusion chromatography, and ultracentrifugation. One possible downside of the protocol is that it includes an overnight step with polyethylene glycol (PEG), which makes the process take longer. However, this step also allows precipitation at lower centrifugation speeds, which can be achieved with an ordinary lab bench-top centrifuge. The isolated UEVs can be feasibly used in various downstream analyses and applications. The UEVs isolated can be applied to proteomic studies such as mass spectroscopy, flow cytometry and ELISA. We also envision our method to be suitable for direct spectroscopic methods such as Raman spectroscopy. Specifically, it has the potential to serve as a starting point for future advances in liquid biopsies and other forms of non-invasive diagnostics.

## Ethics statements

The study was approved by Apollo Hospitals Internal Ethics Committee (IEC) with protocol no. CMBRC/2014/005 and IEC application no 114.

## CRediT authorship contribution statement

**Anula Divyash Singh:** Conceptualization, Methodology, Formal analysis, Software, Writing – original draft. **Sreekanth Patnam:** Validation, Software, Data curation. **Anisha Manocha:** Resources, Investigation, Data curation. **Leena Bashyam:** Resources, Investigation, Data curation. **Aravind Kumar Rengan:** Supervision, Resources. **Manda Venkata Sasidhar:** Writing – review & editing, Supervision, Resources, Funding acquisition, Project administration.

## Declaration of Competing Interest

The authors declare that they have no known competing financial interests or personal relationships that could have appeared to influence the work reported in this paper.

## Data Availability

Data will be made available on request.
